# Supplementary material for: Presynaptic Boutons That Contain Mitochondria Are More Stable
Source: Front Synaptic Neurosci. 2020 Jan 10;11:37. doi: 10.3389/fnsyn.2019.00037 (PMC6966497; doi:10.3389/fnsyn.2019.00037)
Supplement: Supplementary file 1 [file Data_Sheet_1.PDF]

SUPPLEMENTAL INFORMATION

Presynaptic boutons that contain mitochondria are more stable

Robert M. Lees, James D. Johnson, Michael C. Ashby

Supplementary Figures:

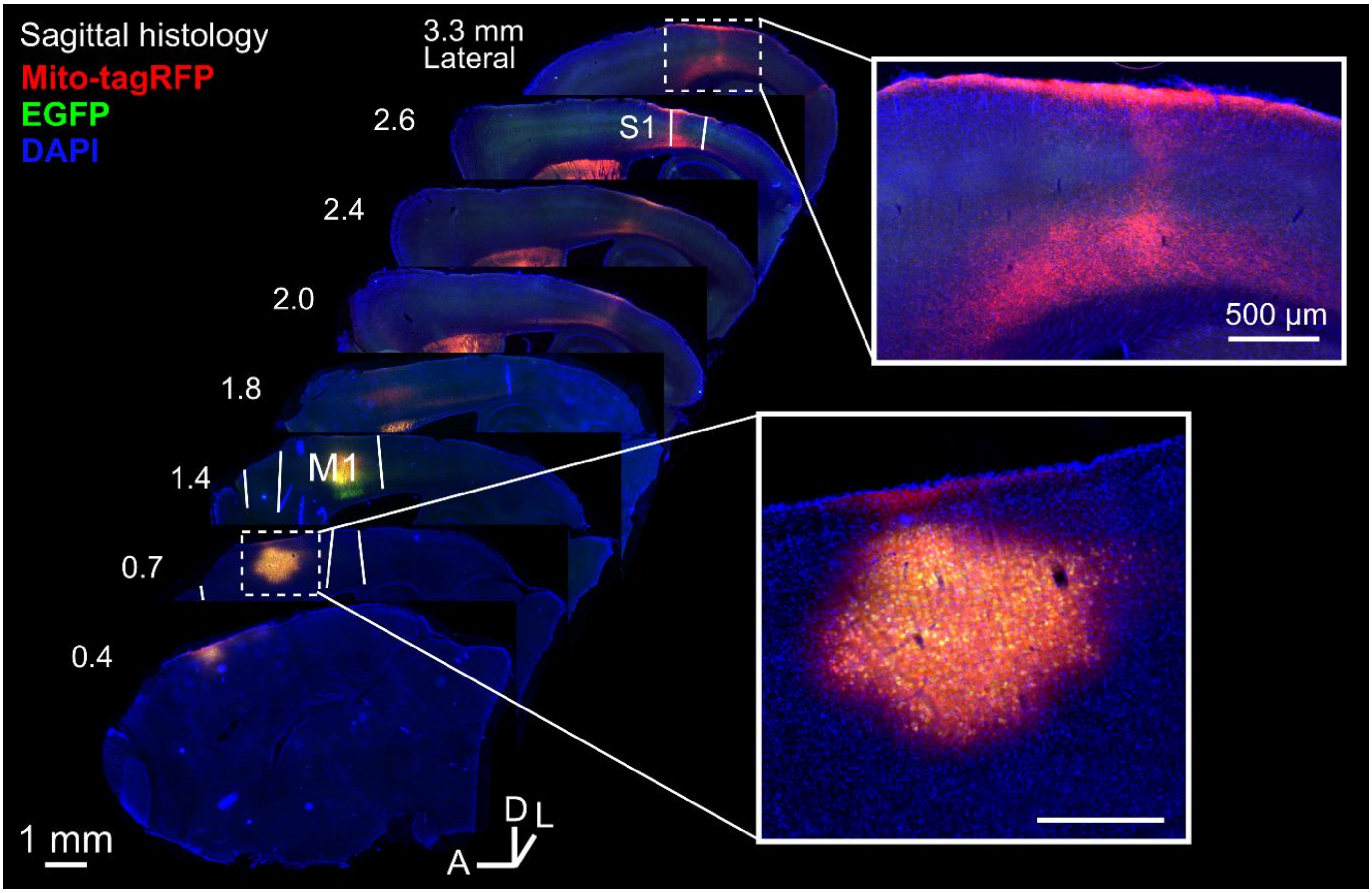

Figure S1 Ipsilateral motor-somatosensory axonal projections. Widefield images of a sagittal series of histological brain slices shows the viral injection site (*bottom inset*) between primary and secondary motor cortex (M1 and M2). Labelled cells projected their axons to subcortical areas as well as through deep cortical layers and ultimately arborizing in superficial layers of somatosensory cortex (S1).

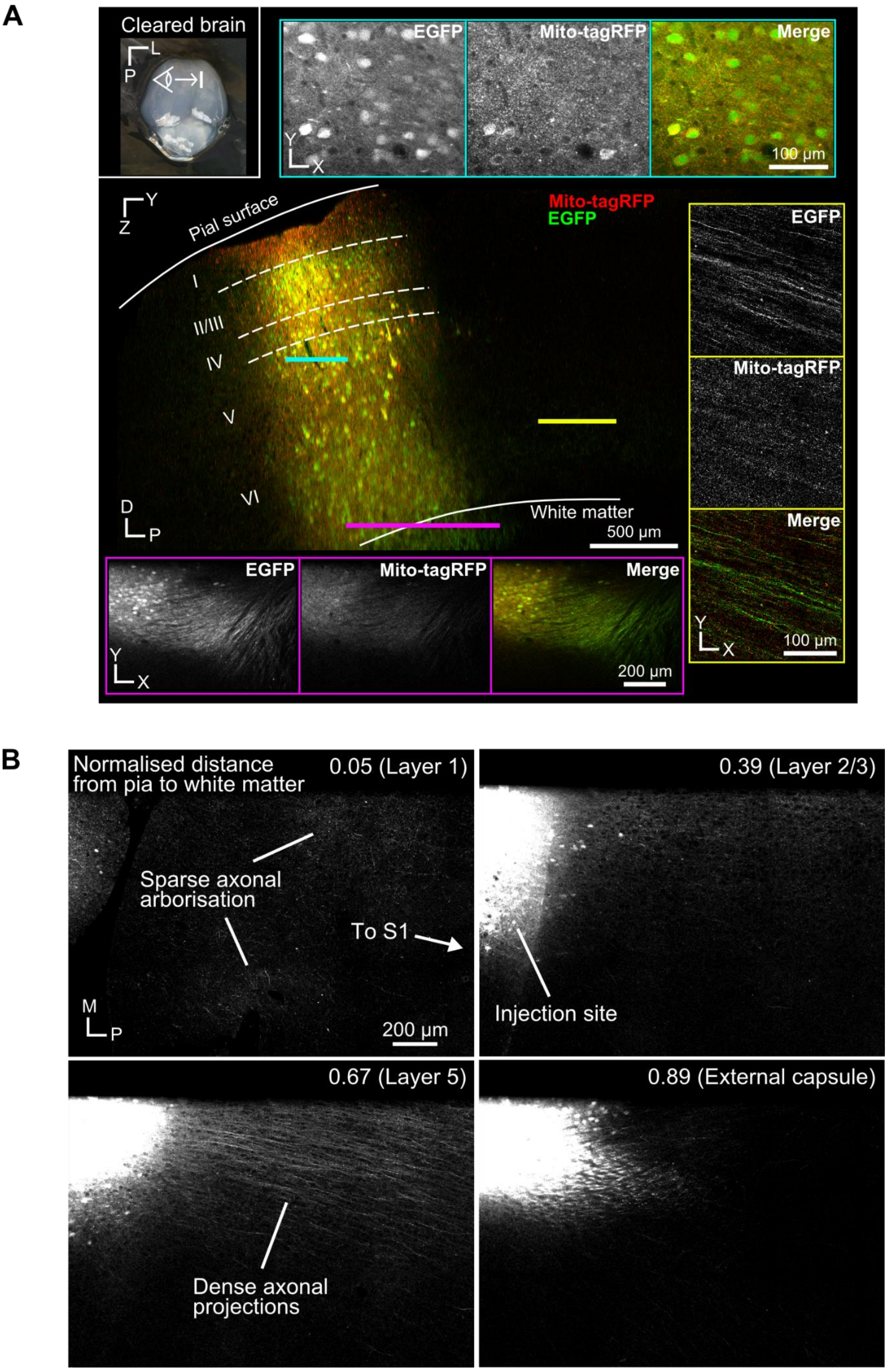

A
Cleared brain
L
P
EGFP
Mito-tagRFP
Merge
Y
X
100 µm
Y
Z
Pial surface
Mito-tagRFP
EGFP
I
II/III
IV
V
VI
D
P
White matter
500 µm
EGFP
Mito-tagRFP
Merge
Y
X
100 µm
EGFP
Mito-tagRFP
Merge
Y
X
200 µm
B
Normalised distance
from pia to white matter
0.05 (Layer 1)
Sparse axonal
arborisation
To S1
M
P
200 µm
0.39 (Layer 2/3)
Injection site
0.67 (Layer 5)
Dense axonal
projections
0.89 (External capsule)

Figure S2 Axonal projections leaving motor cortex. (**A**) (*top-left inset*) low magnification photo of a cleared brain in PBS with the sagittal plane of 2P imaging indicated. (*centre*) 2P image acquired at the viral injection site (sagittal plane). Coloured horizontal lines correspond to inset images with orthogonal imaging planes (horizontal plane). 2P images show the injection site (*top-right inset*), white matter projections (*bottom inset*) and layer V/VI projections (*right inset*). D = dorsal, P = posterior, L = lateral, roman numerals indicate estimated cortical cell layers. (**B**) Horizontal plane through the injection site in cleared brain. (*top-left*) Sparse axonal arborisations in layer 1 of motor cortex. (*top-right*) Over-saturated image showing the injection site at layer 2/3 with sparse axonal arborisation. (*bottom-left*) Dense axonal projections without arborisation emerging from infragranular layers. (*bottom-right*) Axonal projections enter the white matter for subcortical targets. These images indicate that most axonal projections towards somatosensory cortex emerge from layer V/VI. The number in the top-right indicates the normalised distance to the white matter from the pial surface of the cortex. M = medial, P = posterior.

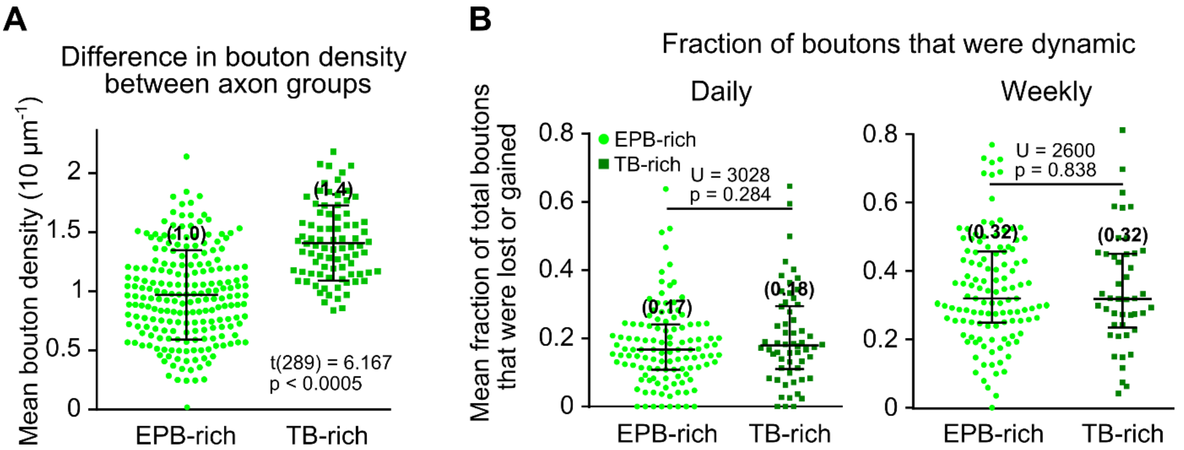

Figure S3 Structural differences along axonal segments were not indicative of bouton turnover (**A**) Bouton density (mean total density across time) was significantly higher for TB-rich axons compared to EPB-rich axons (mean 1.4 ± 0.3, 1 S.D. and 1.0 ± 0.3 per 10 µm, respectively; independent t-test, $p < 0.0005$). Error bars ± 1 S.D. Mean indicated in brackets above error bars. (**B**) The fraction of boutons that were dynamic (either lost *or* gained) was not different between EPB-rich and TB-rich axons (mean fraction across daily intervals, 17% and 18%, or weekly intervals, 32% and 32%, respectively; Mann-Whitney U test, $p > 0.05$). Error bars = inter-quartile range (I.Q.R.). Median indicated in brackets above error bars.

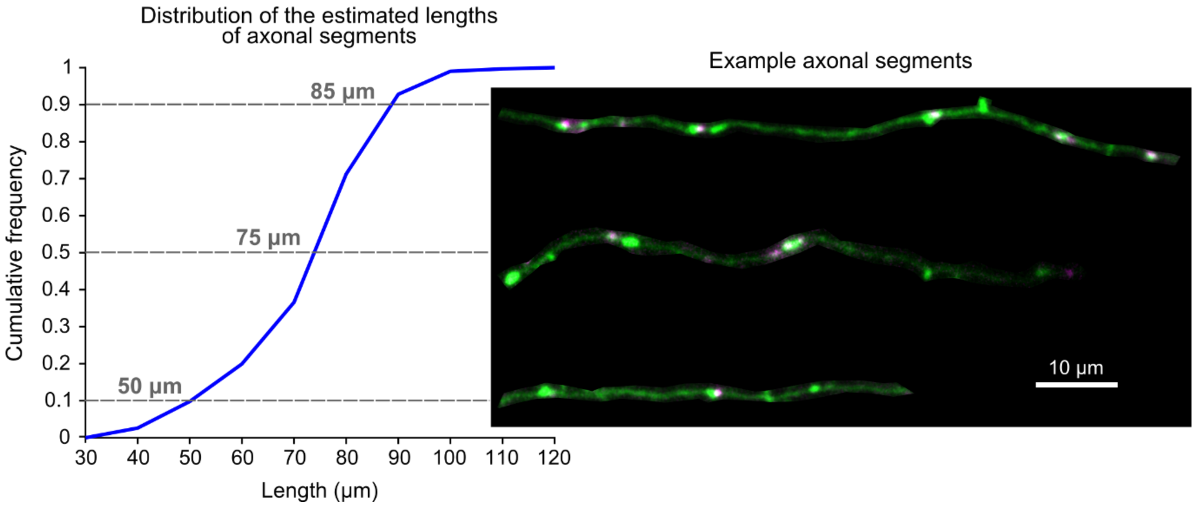

Figure S4 Length distribution of sampled axonal segments. For this study, axonal segments were sampled from 74 x 74 x 30 μm (x, y, z) stacks of 2P images. Each axonal segment length was estimated from a manual tracing made on a 2D projection of each stack (*right*; axons from different fields of view are cropped for presentation). The distributions of estimated axonal segment lengths shows that the median length was 75 μm, with 90% of the sample axonal segments being between 50 and 85 μm (indicated by grey dashed lines).

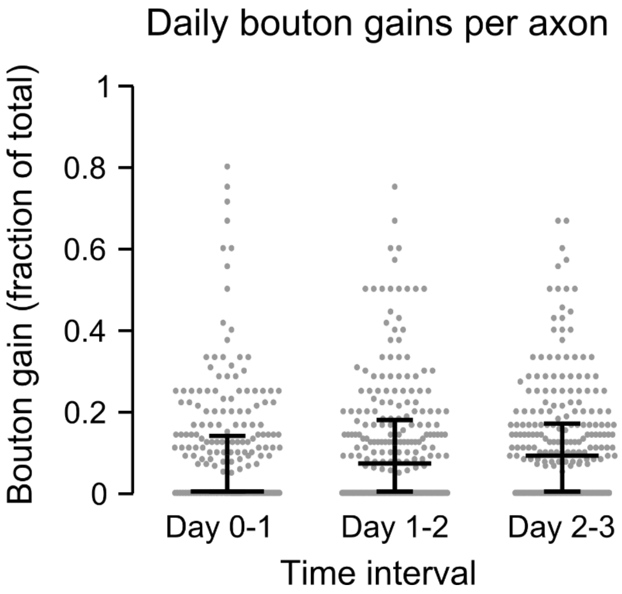

Figure S5 Daily bouton gains as a fraction of the total number of boutons. Over daily intervals the average fraction of boutons gained was 0% (0-14%, I.Q.R.), 8% (0-18%) and 9% (0-17%) (median of the population of axons, n = 249 axons).

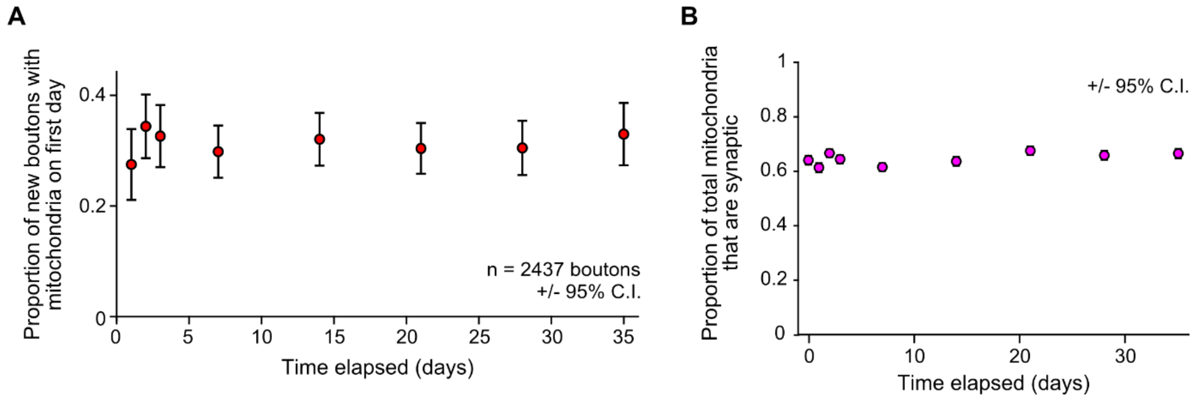

Figure S6 Mitochondrial localisation to presynaptic terminals is consistent for the entire imaging paradigm. (**A**) New boutons formed at every imaging timepoint had the same likelihood of having a mitochondrion nearby, suggesting that increased mitochondrial localisation to long-lived boutons (Figure 4C) is a function of bouton age and not time alone. (**B**) Mitochondria were also no more likely to localise to presynaptic terminals throughout the imaging paradigm.

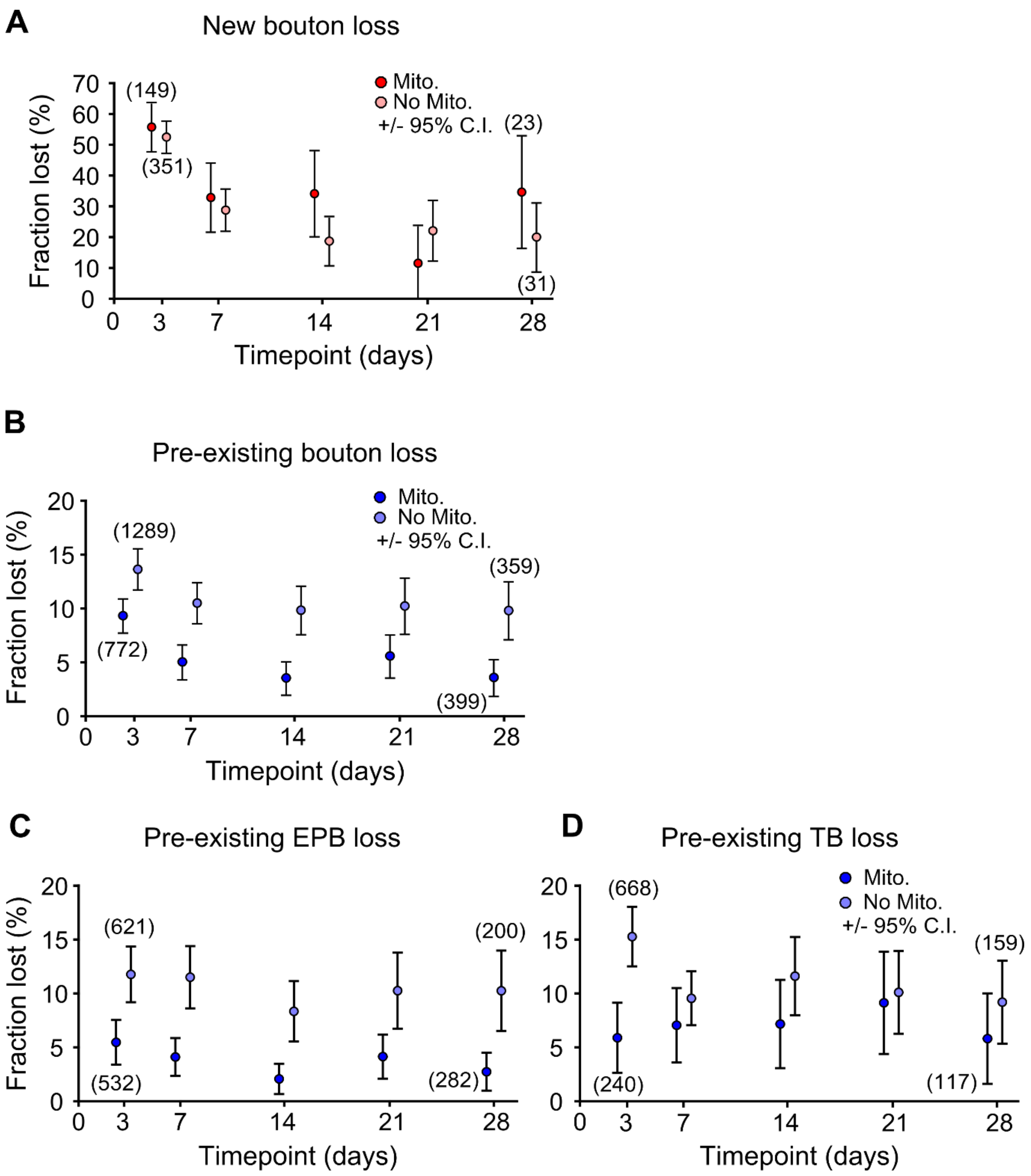

Figure S7 Relationship between mitochondrial presence and bouton loss across time. The number of boutons lost at each timepoint was calculated with relation to the presence of mitochondria, comparisons were made using all the boutons present at each timepoint. (**A**) There was no consistent relationship between mitochondrial presence and bouton loss for newly-formed boutons across time. (**B**) Pre-existing boutons were consistently 30-50% less likely to be lost when mitochondria were localised nearby. (**C-D**) The difference in bouton loss was mainly due to a relationship between

mitochondria and EPBs (C), rather than TBs (D). Numbers in brackets indicate the number of boutons in each group (with or without mitochondria) at the start and end of the experiment. The two groups are plotted either side of each imaging timepoint for easier visualisation of error bars.

Table S1. Spearman's rank correlation at each timepoint to assess the correlation between mitochondrial density and the fraction of dynamic boutons. Weak and non-significant correlations between bouton dynamic fraction and mitochondrion-to-bouton ratio or mitochondrial density were seen. R_S = Spearman's rank correlation, n = 196 axons over all weekly intervals, n = 224 over all daily intervals.

**Mitochondrion-to-bouton ratio**				**Mitochondrial density**			
**Daily**		**Weekly**		**Daily**		**Weekly**	
R_s	**p-value**	R_s	**p-value**	R_s	**p-value**	R_s	**p-value**
0.101	0.133	0.024	0.733	0.012	0.857	0.005	0.944
0.123	0.066	0.033	0.646	-0.013	0.843	-0.079	0.273
0.063	0.348	0.100	0.164	-0.050	0.457	-0.020	0.779
		0.066	0.357			-0.100	0.164
